# Impaired audio-visual associations in dyslexia: evidence beyond linguistic processing

**DOI:** 10.1038/s41539-025-00382-7

**Published:** 2025-12-17

**Authors:** Angela Pasqualotto, Aaron Cochrane, Paola Venuti, Daphne Bavelier, Irene Altarelli

**Affiliations:** 1 Department of Education and Learning, University of Applied Sciences and Arts of Southern Switzerland (SUPSI), Locarno, Switzerland; 2 Faculty of Psychology and Education Sciences (FPSE), University of Geneva, Geneva, Switzerland; 3 Department of Cognitive and Psychological Sciences (CoPsy), Brown University, Providence, RI USA; 4 Department of Psychology and Cognitive Science, University of Trento, Trento, Italy; 5 Campus Biotech, Geneva, Switzerland; 6 Dipartimento di Medicina dei Sistemi, Università degli studi di Roma Tor Vergata, Rome, Italy; 7 LaPsyDÉ, Université Paris Cité, CNRS, Paris, France

**Keywords:** Human behaviour, Dyslexia

## Abstract

Audio-visual (AV) associations are central to many aspects of behavior, including the initial steps of learning to read. The acquisition of AV pairings has been explored in individuals with varying literacy skills, including children with developmental dyslexia. Most previous studies examined performance in AV associative tasks looking at the pairings between linguistic auditory material and visual stimuli, thus confounding AV learning with phonological and/or verbal abilities. In the present study, we introduce an AV learning paradigm relying on non-linguistic auditory stimuli and novel visual shapes. We fit trial-by-trial performance and compare the response patterns of 52 Italian-speaking children with developmental dyslexia (DD) with those of age-matched (N = 54) and of younger, reading-matched (N = 51) typically-developing children. All groups showed increasing accuracy across trials, but children with DD learned less efficiently than their peers. These findings suggest that difficulties in forming AV associations through repeated exposure may underlie dyslexia, even when linguistic demands are minimized.

## Introduction

Audio-visual (AV) associative learning, the process by which auditory and visual stimuli are linked together through repeated exposure^1^, is central to many aspects of cognitive development and is particularly crucial for the initial steps of reading acquisition. Yet its potential contribution to neurodevelopmental disorders remains poorly characterized, limiting our understanding of how AV learning difficulties may affect broader cognitive trajectories.

Learning to read involves the acquisition of arbitrary associations between visual symbols (graphemes) and speech sound units (phonemes). This process depends on two key components. First, it requires well-specified unimodal representations, such as phonemic and visual representations that are robust against acoustic variations induced by different speakers, dialects, or contexts^2^, and against visual disparities entailed by different letter cases or fonts, respectively. Second, it relies on efficient associative learning skills in order to build lasting pairings between those auditory and visual stimuli. Therefore, individual variability in the ability to learn audiovisual associations may result from differences in unimodal processing, in associative learning skills, or from the interaction of the two.

Interestingly, research in typically-developing children has revealed that performance in AV learning predicts reading skills over and above classical reading-related skills such as phoneme deletion^3–6^. Additionally, associations have been found between AV learning abilities and domain-general skills like verbal working memory, interference control, and auditory attention^7,8^. However, whether these domain-general abilities mediate the relation between AV learning and reading skills is yet unclear.

AV associative learning processes have also been explored in individuals with varying reading proficiency levels, including in children and adults with developmental dyslexia (DD)^9^. DD is characterized by persistent reading difficulties despite adequate intelligence and educational opportunities^10^, affecting an estimated 3–10% of the population. According to the predominant theory of DD etiology, severe and persistent reading impairments in readers with DD arise from an underlying phonological deficit—that is, a difficulty in representing, accessing, and/or manipulating the sound structure of spoken language (referred to as phonology)^11^. This deficit manifests in various tasks, such as phonological awareness (e.g., identifying or manipulating sounds), verbal short-term memory, and rapid verbal retrieval tasks^12^. While altered phonological development and impaired phonological skills have been recognized as key pathways leading to dyslexia^13^, numerous studies suggest that the etiology of dyslexia may be multifactorial rather than purely phonological^14–17^. Firstly, some domain-general cognitive deficits have been linked to DD^17–21^, particularly deficits in visual attention^18,21–23^ (for recent reviews, see refs. ^24–26^), in working memory, and in cognitive control^19,20,27,28^. Importantly, causal evidence suggests that attentional mechanisms in DD can be enhanced through interventions such as action video game training and non-invasive brain stimulation^29,30^. Secondly, there is evidence indicating that dyslexic readers exhibit specific deficits in cross-modal integration^9,31,32^ and in forming associations between graphemes and phonemes^33,34^. Difficulties in associating familiar letters and speech sounds have also been reported in kindergarten children at familial risk of DD^35^.

AV associative learning studies have shown that individuals with DD learn significantly less well than their typically-developing peers in these AV tasks. A number of studies have highlighted that, after initial training to a high level of performance, children and adults with dyslexia are less accurate in subsequently recalling learned AV associations, especially when the pairings involve linguistic stimuli^36–39^ (but see ref. ^40^ for an exception where no group difference was found despite linguistic materials). Differences have also been found in assessments of generalization beyond initially trained tasks. Aravena et al.^40,41^ asked children to learn AV correspondences among unfamiliar symbols (Hebrew letters) and familiar native phonemes (Dutch) and found that individuals with dyslexia performed worse than their typically-developing peers on reading tasks involving this new writing system. Studies examining the progression over time of learning AV associations are less common; yet, Guerra et al.^8^ found a divergence in learning progress between children with dyslexia and their peers, with the former showing lower accuracy towards the end of the task. Finally, a number of studies have tried to disentangle unimodal versus AV associative learning abilities. Messbauer and de Jong^37^, for instance, showed that children with dyslexia exhibit deficits in cross-modal associative learning tasks involving verbal stimuli, such as words or nonwords; yet perform comparably to age- and reading-matched controls on unimodal visual–visual associative learning tasks. Litt and Nation^36^ have argued that verbal output is instrumental in revealing group differences between children with dyslexia and their peers, and that phonological processing, rather than AV associative learning, is hindered.

The proper interpretation of these results needs to be cautioned by the high variability in methodology across studies. This variability is found at the levels of (i) the demands of the paradigms employed (e.g., examining the processing of congruent pairs in letter-speech sound priming tasks vs. asking participants to pick one element of a pair after presentation of the other element—thus loading more or less heavily on skills such as phonological processing, working memory and speech production), (ii) the types of stimuli used (auditory ones such as phonemes, syllables, words or nonwords; visual ones such as pictures or unfamiliar shapes), (iii) the difficulty of the tasks employed (as can be estimated, for instance, from the number of associations to be built and retained) and (iv) the phases of learning assessed (encoding, recalling, generalization). These differ widely from one study to the next, rendering systematic interpretation difficult. Moreover, to the best of our knowledge, all of the studies comparing individuals with DD to a control population have used linguistic AV mappings, asking children to map language sounds to visual input, thus introducing confounds between AV learning per se and the phonological (or more generally verbal) abilities of the learner. Finally, because the linguistic stimuli employed are typically taken from the spoken language of the learners, asymmetries in the familiarity of the unimodal stimuli used (i.e., greater familiarity with the auditory elements of the pairs compared to the visual elements) are usually introduced in these paradigms.

Provided that the majority of children with dyslexia present impairments in phonological abilities, either isolated or combined with other cognitive deficits^42^, a key step towards reaching a better understanding of the link between associative AV learning and reading skills is to test non-linguistic AV associative learning in children with DD, contrary to most previous literature. In that case, if less efficient learning is found in children with dyslexia compared to a typically-developing population, despite the absence of linguistic stimuli potentially calling upon phonological skills, then difficulties in AV associative learning may be interpreted as a putative core deficit of DD. Conversely, if a non-linguistic AV associative learning task reveals no difference in learning between children with and without dyslexia, then previous learning-related results in dyslexia may be interpreted as a consequence of poor phonological abilities, rather than an AV learning impairment per se.

**Fig. 1:**
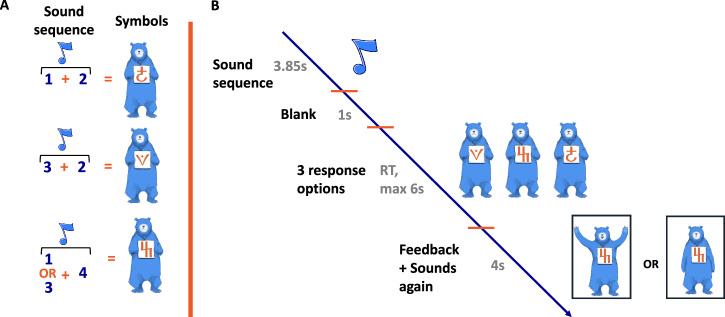
Task design. **A** Four to-be-learnt audio-visual pairs. Children learned to associate four specific sound combinations (1 + 2, 3 + 2, 1 + 4, 3 + 4) with three different visual symbols (bears). In combinations 1 + 2 and 3 + 2, both sounds in the sequence are important for predicting the corresponding symbol, whereas in combinations 1 + 4 and 3 + 4, only the last sound is necessary. **B** Trial Structure. Each trial began with a combination of auditory stimuli (played without an inter-stimulus interval), accompanied by a small musical note displayed on the screen. This was followed by a blank screen, after which the response options appeared. After the child’s response, visual feedback was provided (e.g., a bear with arms raised indicated a correct response), along with the corresponding sounds being played again.

To address the limitations of previous studies, we adapted a non-linguistic AV learning paradigm previously used to study learning in adults^43^. In this paradigm, participants were tasked with learning arbitrary associations between unfamiliar sounds (e.g., vaguely reminiscent of a siren, a rolling object, or an uprising melody, but not straightforwardly nameable) and novel visual symbols adapted from the Bamum alphabet (see Fig. 1). Therefore, both auditory and visual stimuli to-be-paired are equally novel and elicit limited reliance on phonological and/or verbal skills. To make the task suitable for children, we gradually increased complexity by delaying the introduction of some audiovisual pairings. While the entire stimulus set included four combinations of sounds, children were exposed to two combinations in the first block, to a third combination in the second block, and to all four combinations from the third block onwards (see Table 1).

**Table 1:** Scaffolding structure

Block 1	Sound 1 + 2—symbol A
	Sound 3 + 4—symbol B
Block 2	Sound 1 + 2—symbol A
	Sound 3 + 4—symbol B
	*Sound 3* + *2—symbol C*
Block 3–6	Sound 1 + 2—symbol A
	Sound 3 + 4—symbol B
	Sound 3 + 2—symbol C
	*Sound 1* + *4—symbol B*

In block 2, a novel sound pair is introduced, making both sounds crucial for predicting the corresponding visual symbol. In block 3, a novel sound pair is introduced that matches an already associated symbol, violating the one-to-one mapping existing so far.

To draw inferences about (a) group-level differences in learning, (b) individual rates of learning, and (c) the effects of introducing novel stimuli partway through learning, we applied a fully continuous-time model to analyze trial-by-trial accuracy data^43–45^. Our primary focus was on identifying individual learning trajectories using all available trials (*n* = 72) to better characterize the learning process itself, rather than focusing on a single trajectory point or a putatively stable ending outcome. Trajectories of performance were parameterized as having starting accuracies, asymptotic accuracies (i.e., accuracy expected with extended training), and a time constant defining the rate of change from start to asymptote^44,46^. This approach aimed to provide a clearer understanding of the learning dynamics over time. This aligns with the dynamic framework of reading acquisition proposed by Bonte and Brem^47^, which emphasizes the importance of capturing short-term learning trajectories across multiple stages and processes as indicators of long-term reading development.

We compared the performance of Italian-speaking children with DD to typically developing controls. Participants were recruited over two waves, with the preregistered Wave 2^48^ replicating the results of Wave 1. The combined sample of 157 children was distributed across three groups: children with DD (*N* = 52), age-matched typically-developing controls (*N* = 54), and younger reading-matched controls (*N* = 51). This design allowed for robust comparisons across different developmental stages and reading abilities. Finally, we examined the relations between AV learning outcomes and various linguistic and cognitive functions, including phonological awareness, rapid automatized naming (RAN), and executive functions.

The current study is therefore well-equipped to address the question of whether children with DD display similar or diverging associative AV learning performance from their typically-developing peers (one group matched for age and another matched for reading ability), when confounds from the familiarity of the stimuli and their phonological/verbal properties are avoided. We assess the following hypotheses:

1. *AV associative learning*: We expect that children with DD will show impaired AV learning performance compared to their typically-developing age-matched and reading-matched peers, even when the task uses non-linguistic stimuli. If this hypothesis is confirmed, it would suggest a cross-modal learning deficit in DD that goes beyond difficulties in phonological or verbal processing^40,41^.
2. *Reading skills and AV learning*: We expect the performance of our best readers (age-matched controls) to be higher than that of the DD group, but also that of the reading-matched controls. If confirmed, this would indicate that, once typical reading ability has been achieved, then reading ability itself may facilitate subsequent AV associative learning performance above and beyond age alone. This is in line with a mutualistic view of cognitive development where progress in one ability is interrelated with progress in another^49^.
3. *Phonological skills and AV learning*: In line with hypothesis 1 above, phonological skills may not be related to learning in our task (matching the observations of Pasqualotto et al.^43^ in adult populations from multiple linguistic backgrounds). Thus, we do not expect phonological awareness and our AV learning to be related, neither within the group of children with DD nor within each of the control groups.

In our experimental design, children listened to two sounds presented back to back and then selected the visual symbol associated with that sound pair. The stimuli are built such that for two of the sound pairs, the last sound is uniquely diagnostic of the visual symbol (easier associations), while for the other two pairs, neither the first nor the last sound can be used, since both sounds together are uniquely diagnostic of the visual symbol associated with them (harder associations; see Fig. 1A).

In addition, as mentioned above, the AV pairs were introduced in a scaffolded manner, with children being first exposed to two totally non-overlapping sound pairs in Block 1, allowing children to succeed in the AV mapping, whether they considered the pair of sounds together or just the last sound. Then, in Block 2, the added AV pair enforced taking into account both sounds for AV mapping. Finally, in Block 3, a novel AV pair was introduced. While presenting an easy-to-learn mapping (which could be done based on the last sound only), it nevertheless broke the principle of one pair of sounds being associated uniquely with one visual item (because now two sound pairs were associated with a single visual item). This scaffolding introduces unique and different learning challenges both at the start of Block 2 and of Block 3 (see Table 1).

## Results

We first present the learning-related results for the two waves combined, followed by the results for each wave separately. Means and 95% credible intervals of posterior distributions are reported from nonlinear generalized Bayesian mixed-effects models.

### Learning

**Fig. 2:**
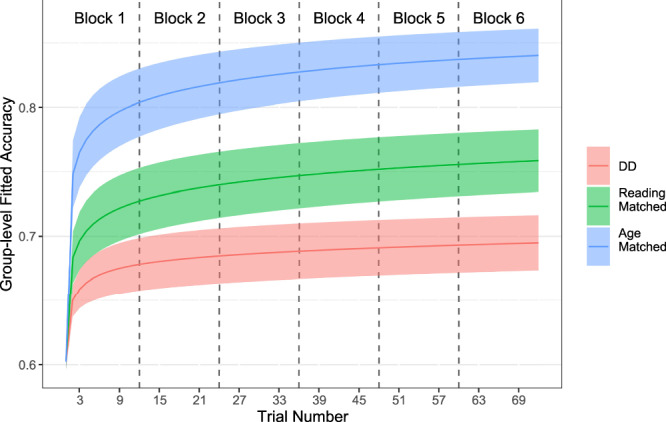
Learning trajectories of each group, estimated using a model combining across both waves. Decreased accuracy upon the introduction of new stimuli in Block 2 and Block 3 is controlled for in our nonlinear hierarchical model of learning, and is not shown here (see Figs. S1 and S2).

Across both waves, the combined results revealed that both the age-matched and reading-matched groups demonstrated higher asymptotic accuracy in the learning task than children with DD, as shown in Fig. 2 (age-matched vs. DD mean = 0.505, CI_95_ = [0.37, 0.64]; reading-matched vs. DD mean = 0.283, CI_95_ = [0.146, 0.43]; all comparisons of asymptotes are on log-odds scales; see Table 2). Further, age-matched children achieved higher asymptotic accuracy than reading-matched children (age-matched vs. reading-matched mean = 0.223 CI_95_ = [0.12, 0.32]).

**Table 2:** Results of the nonlinear mixed-effects learning model fit to both waves together

	Estimate	l-95% CI	u-95% CI	reliable
**Learning**				
Time-to-learn (DD)	0.622	−1.997	3.201	N/A
Time-to-learn (Age-Matched—DD)	−3.092	−5.784	−0.579	*
Time-to-learn (Reading-Matched—DD)	−0.755	−3.373	1.757	
Asymptote (DD)	−0.041	−0.272	0.173	N/A
Asymptote (Age-Matched—DD)	0.505	0.370	0.640	*
Asymptote (Reading-Matched—DD)	0.283	0.146	0.430	*
**Introduction of a novel stimulus**				
DD: Block 2 offset	0.056	−0.082	0.184	
Age-Matched: Block 2 offset	−0.039	−0.155	0.062	
Reading-Matched: Block 2 offset	−0.163	−0.300	−0.039	*
DD: Block 3 offset	−0.063	−0.197	0.077	
Age-Matched: Block 3 offset	−0.348	−0.470	−0.222	*
Reading-Matched: Block 3 offset	−0.109	−0.243	0.021	

Fixed effects are shown here, with means and CI of posterior distributions.

Age-Matched and Reading-Matched groups’ Rate [log time constant; “time to learn”] and Asymptote estimates are in contrast to the DD’s estimates. Results are presented this way because, for rate and asymptote, there is no objective baseline, so the DD group is the comparison group. For interference, because there is an objective baseline (i.e., 0 change), all groups’ estimates are compared to zero. In this table, no effects of the cohort are presented to simplify the presentation of the results. That is, in this table, all results are controlling for cohort effects and averaging across any differences between cohorts. Full results of fixed-effects estimates, including with effects of cohort, are presented in the SI. Time-to-learn values are reported on a log scale with base 2. All other measures are on an inverse-logistic (log-odds) scale.

In Wave 1 (*n* = 70), we observed that both age-matched and reading-matched groups demonstrated higher asymptotic accuracy in the learning task than children with DD (age-matched vs. DD mean = 0.363, CI_95_ = [0.171, 0.546]; reading-matched vs. DD mean = 0.254, CI_95_ = [0.058, 0.437]; see Fig. S1). In our preregistered Wave 2 (*n* = 87)^48^, we replicated the finding that both age-matched and reading-matched groups demonstrated higher asymptotic accuracy in the learning task than children with DD (age-matched vs. DD mean = 0.706, CI_95_ = [0.541, 0.932]; reading-matched vs. DD mean = 0.28, CI_95_ = [0.136, 0.436]; see Fig. S2). Together, these findings from two distinct waves of data collection suggest that children with DD display poorer audiovisual associative learning skills, even in a task that fully relies on non-linguistic stimuli.

### Learning and relearning as a novel stimulus is introduced

**Fig. 3:**
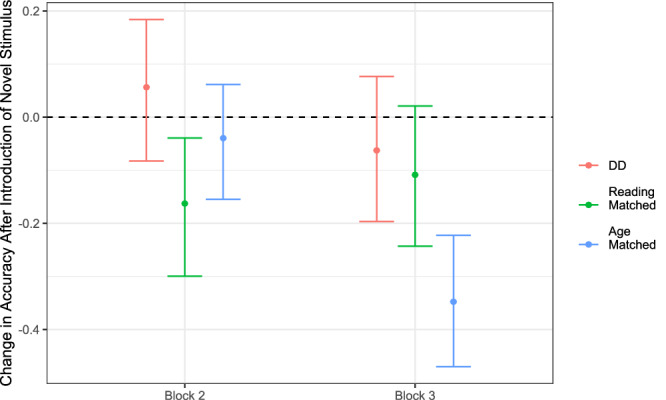
Estimated changes in AV learning task accuracy after the introduction of a novel sound pair combination to be mapped on a novel visual symbol in block 2 (left) and a novel sound pair combination to be mapped on an already-mapped visual symbol in block 3, violating the regularities learned so far (right). These changes in accuracy were estimated jointly with the overall learning trajectories and represent deviations from those trajectories. Note. In block 2, the reading-matched participants were the only group showing a reliable change (i.e., decrease) in performance, while in block 3, the age-matched participants were the only group showing a reliable decrease in performance.

The introduction of a new stimulus, in which previously used sounds are recombined to match a new visual form, in Block 2, led to a reliable decrease in accuracy only among the reading-matched controls. While this effect had not been reliable in the results from just Wave 1 (mean = −0.057, CI_95_ = [−0.248, 0.097]), it was evident in Wave 2 (mean = −0.295, CI_95_ = [−0.482, −0.113]), as well as when considering both waves together (mean = −0.163, CI_95_ = [−0.300, −0.039]; see Fig. 3 and Table 2).

In Block 3, the new stimulus was also made of a recombination of known sounds (namely, sound 1 and sound 4) but now mapping to an already associated visual symbol (symbol B); here, the introduction of this new stimulus tended to decrease performance in all groups. Yet, the decrease was reliably different from zero change only in the age-matched group (mean = −0.348, CI_95_ = [−0.47, −0.222]; see Fig. 3 and Table 2). This effect was visible considering both waves together, as well as in Wave 1 (mean = −0.359, CI_95_ = [−0.537, −0.184]) and Wave 2 (mean = −0.329, CI_95_ = [−0.499, −0.16]) separately. Additionally, when comparing groups’ decreases, the age-matched group’s decrease was larger than that of the DD group (mean = −0.285, CI_95_ = [−0.462, −0.105]) and of the reading-matched group (mean = −0.239, CI_95_ = [−0.417, −0.059]). The observed decrease in the age-matched controls was greater than what would be expected solely from the introduction of one unfamiliar stimulus out of four, which would predict a return to chance-level guessing for that specific stimulus. Such a pattern suggests that the age-matched group may have been more sensitive to the introduction of that novel stimuli association, which temporarily disrupted their performance; however, clear interpretations of these results are obscured by the fact that the age-matched group was already performing much better than either of the other groups by the beginning of block 3 (see Fig. 2). For further information on cohort specific results see Tables S1 and S2.

### Performance in cognitive, reading, and reading-related tasks, as well as their associations with AV learning

**Fig. 4:**
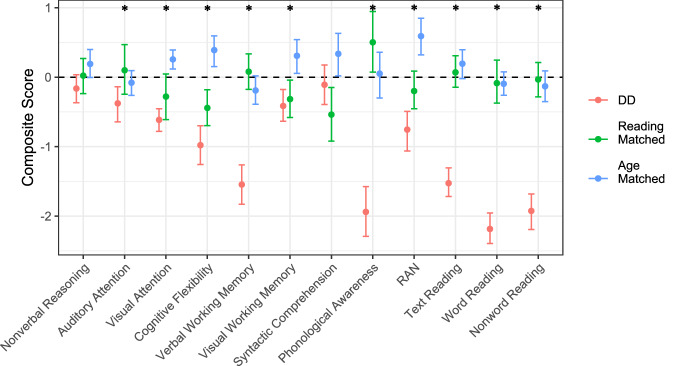
Composite scores for each reading, reading-related, and cognitive measure, combined across waves (for separated plots see Fig. S4). Error bars indicate bootstrapped 95% confidence intervals. Asterisks indicate DD groups’ correlations being reliably different than zero. See “Methods” section for score normalization.

Overall, children with DD consistently showed lower performance compared to both age-matched and reading-matched children in reading, reading-related, and cognitive skills, except for nonverbal reasoning skills and syntactic comprehension (Fig. 4). A performance gap was evident in those competencies that are known to be critical to reading development, such as phonological awareness—as largely reported in previous literature^5,13^.

**Fig. 5:**
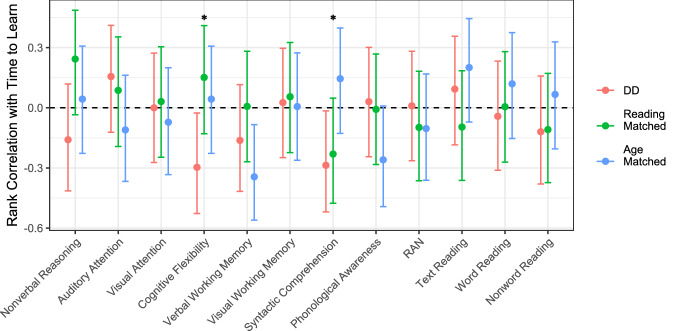
Correlations between each measure and time-taken-to-learn in the AV learning task, combined across waves (for results separated by wave see Fig. S3). Negative correlations indicate more efficient learning as performance increases on the non-learning measure. Error bars indicate bootstrapped 95% CI of rank correlations. Asterisks indicate DD groups’ correlations being reliably different than zero. Each measure was *z*-scored within wave before combining across waves to calculate these correlations.

Further analyses explored the relationship between these measures and time-taken-to-learn in the AV learning task (see Fig. 5; of note, reported correlations are not corrected for multiple comparisons; results for each individual wave are available in Fig. S3). Of note, negative correlations here indicate links between higher task scores and more efficient learning. First, performance in the phonological awareness task did not show a significant correlation with learning in the AV task across any of the groups, in line with what was previously reported in adults^43^. Second, reliable correlations were found with certain non-phonological skills. Notably, better syntactic comprehension was linked to shorter time-taken-to-learn in both the reading-matched and DD groups. In the DD group, greater cognitive flexibility showed a significant correlation with faster learning. Greater verbal working memory was linked to faster learning in the age-matched group, as it has previously been shown in adults. These results suggest that these various skills may support learning efficiency within these respective groups and align with our hypothesis, supporting the expectation that phonological skills would not be directly related to learning efficiency in our non-linguistic AV learning task.

## Discussion

This study explored AV associative learning using a carefully designed non-linguistic task, accessible to children across a range of literacy abilities, including reading-matched participants as young as 8 years of age. Our paradigm relies on non-linguistic auditory stimuli paired with novel visual symbols, which minimizes the influence of phonological skills and of familiarity with the visual stimuli. The task’s scaffolded design provides a gradual introduction of stimulus pairs, allowing children to progress in stages and adapt to new associations over time. By capturing trial-by-trial accuracy, the task revealed both group and individual differences in learning rates, showcasing how children adapt to its learning demands and respond to the introduction of new stimuli. Importantly, our results are strengthened by data collection across two waves, with the preregistered second wave^48^ replicating the findings of the first. This two-wave design provides robust evidence for the observed patterns in AV learning across different reading abilities.

Our findings reveal that typically-developing children as well as children with DD demonstrated progress across the AV task. However, children with DD consistently exhibited lower accuracy, suggesting difficulties in AV associative learning that extend beyond phonological processing deficits. Furthermore, our results highlight a potential link between literacy skills and AV learning proficiency; children with stronger reading skills tended to reach higher asymptotic accuracy. This pattern could align with a mutualistic model of cognitive development, wherein advances in the acquisition of one ability, such as reading, may support and interact with other learning processes like AV associations’ learning^47,49^.

The dyslexia literature has been marked by a controversy around whether AV learning difficulties in children with DD might be largely due to issues in processing linguistic materials. Several studies have favored this option, claiming that weaknesses in AV associative learning in DD are strongly linked to—if not fully determined by—the verbal demands of the task^36,37,50,51^. For instance, Litt and Nation^36^ compared associative learning in a number of conditions (namely, visual–verbal, verbal–verbal, verbal–visual, and visual–visual) between children with DD and age-matched controls. Their results showed poorer learning in DD only when the task required verbal output; they also highlighted that difficulties in the visual–verbal condition were fully accounted for by phonological form learning. Interestingly, our results directly speak to that controversy and clearly go against the verbal processing deficit interpretation, given that our AV learning paradigm employs unfamiliar non-linguistic auditory stimuli and thus minimizes the influence of linguistic skills. Not only did children with DD differ from age- and reading-matched controls in AV learning in our task across two distinct experiments (Wave 1 and Wave 2), but we also found no correlation between phonological awareness skills and learning in our AV task across any of the groups.

Our findings, therefore, motivate a reinterpretation of the difficulties encountered by DD individuals in AV learning, especially in terms of the subprocesses that could be affected. To form durable AV associations, clear representations of both the auditory and the visual stimuli need to be available. Then, the binding itself of newly associated auditory and visual information needs to be coded and maintained, first in working memory. Indeed, forming AV pairs could be considered to rely on the process of binding in working memory, that is, the integration of a number of isolated characteristics into a compound representation^52,53^. Eventually, through multiple repetitions, the bound AV representations are expected to be consolidated in long-term memory^54^. Any of the subprocesses within AV learning could potentially be impaired in children with DD, leading to less proficient learning. Unimodal stimuli to be associated could be represented or manipulated less efficiently by individuals with DD, in particular as regards the auditory stimuli: poorer performance in the discrimination of nonspeech sounds compared to controls has been reported in the past (see ref. ^55^ for a meta-analysis; but see refs. ^56,57^). Some evidence also exists that individuals with dyslexia retain past episodic traces less well and/or integrate them less efficiently over multiple instances, compared to typical readers—a process that, on top of maintenance within a buffer, most probably also requires flexible allocation of attentional resources^58,59^. Domain-general skills are likely candidates for supporting many of the described unobserved steps within AV learning. For instance, previous studies have hypothesized a failure to deploy attentional resources in cross-modal binding in DD^8,59^. A previous study in adults, using a very similar paradigm, found an association between learning performance and verbal working memory, across two separate groups of participants (French-speaking and Italian-speaking participants^43^)—a finding that may also be related to the very structure of our task, where two sounds are presented one after the other. Further characterization of the putative auditory processing difficulties in our task and of the domain-general skills involved in each sub-process of AV learning will be important to gain further understanding of the origins of the differences between more and less skilled readers, as evidenced in the current study. It could also pave the way to intervention studies that may help reveal the causal relationships at play^29^.

While these results highlight the challenges faced by children with DD in AV associative learning, they also raise broader questions about the developmental trajectory of AV learning skills in typical readers. Understanding how the relationship between AV learning and phonological awareness evolves across various stages of reading development is crucial for interpreting the observed differences. In young children, particularly pre-readers, variations in phonological awareness skills appear to be associated with their ability to acquire non-linguistic AV pairings^60^. This is probably due to the fact that both of these competences rest on the ability to extract and integrate redundant auditory and visual information. In the case of phonological awareness, one contributing factor in pre-readers may be their capacity to integrate auditory (oral language) with visual speech cues, such as lip movements. Evidence suggests that children can benefit from such multimodal information, though reliance on lip-reading varies considerably across individuals. The ability to efficiently extract and process audiovisual cues may contribute to the formation of more precise phonological categories, ultimately enhancing phonological awareness. As children progress in their (reading) development, we would expect a shift in the factors influencing AV learning performance. While in pre-readers, AV learning (even non-linguistic) is related to phonological awareness^60^, in proficient readers, it appears to be associated with domain-general cognitive skills, particularly working memory capacity^43^. This pattern suggests a developmental transition: as children become skilled readers, their reliance on phonological awareness for AV learning weakens, and the role of working memory capacity becomes more prominent. This is consistent with findings in adult populations, where we previously demonstrated that working memory capacity, rather than phonological awareness, predicts AV learning performance in both French and Italian speakers^43^.

Further supporting this developmental shift, our comparison between children with DD and reading-matched controls provides additional insight into the role of reading experience in AV learning. While one might hypothesize that AV associative learning inherent to reading acquisition facilitates AV learning (i.e., learning to read acting as “training” to form AV associations), our results show that reading-matched controls still outperformed children with DD, despite being younger. This suggests that AV learning difficulties in DD are not simply a byproduct of their reading skills, as may be expected if proficient reading were to be the main contributor to enhancing AV learning, but may instead reflect a more fundamental difficulty in forming and consolidating cross-modal associations.

The progressive introduction of new stimulus pairs across blocks allowed us to examine how participants adapted to increased complexity in AV learning. Reading-matched children displayed a temporary decrease in performance with the new pairing introduced in Block 2, which may have been due to younger learners struggling with the cognitive load associated with adapting to novel associations. In contrast, more advanced learners (i.e., age-matched controls) appeared to easily overcome the reconsideration of the space to be learned after the introduction of a new stimulus in block 2, but were more challenged by the new item being introduced within block 3. In this group, block 3 showed a reduction in accuracy that brought them to comparable levels of accuracy with respect to the DD group (see Figs. S1 and S2 for visualizations of accuracy over time by group). While the difficulties observed in our learning task appear to be especially pronounced in children with DD, as reported above, the performance decline observed in age-matched controls suggests they may have entertained a stronger model of the state space (i.e., an internal representation of the possible mappings between sounds and symbols), which the new stimulus introduced in Block 3 violates. Such a disruption likely requires cognitive resources to adjust to, in line with their overall time to learn, being linked to verbal memory capacities. Block transitions thus offered a natural probe of learning robustness, highlighting how children adapted to discontinuities in task structure. The disruption and recovery patterns from the novel stimulus introduced at Block 3 may be particularly relevant for understanding the adaptability required for reading development, where proficient readers must navigate and adjust to unexpected changes (e.g., learning new orthographic rules).

The present work offers a valuable foundation for understanding domain-general mechanisms underlying AV learning, particularly in relation to DD. While our correlational findings offer insights into the cognitive and linguistic profiles associated with AV learning, they should be interpreted with caution, given the small sample sizes and multiple associations tested without correction. Larger studies are needed to confirm these patterns and clarify the role of cognitive abilities—such as working memory—in AV learning, especially in children with DD.

Crucially, our task was designed to isolate AV learning from phonological skills, and the absence of a significant correlation with phonological awareness supports its non-linguistic, domain-general focus. By introducing novel stimuli that tap both the formation of new associations and flexibility in updating state-space configurations, our task captures key components of associative learning. At the same time, some additional design-related limitations should be acknowledged. Although our design aimed to reduce reliance on phonological skills by using unfamiliar environmental sounds, individual differences in familiarity or affective associations with the stimuli may have introduced variability in performance. Moreover, we progressively added new pairings across blocks, which could, in principle, affect the unfolding of AV acquisition. However, we deliberately exploited these block transitions as a diagnostic probe of learning robustness, offering insights into how children adapt to discontinuities. Finally, the asymmetry between auditory and visual familiarization phases may have led to stronger encoding of visual relative to auditory inputs, as only the former involved an active task. Future studies could address this by implementing an active auditory discrimination or matching task to balance engagement across modalities.

Beyond addressing these design considerations, applying this paradigm to younger children, with or without familial risk of dyslexia, may shed light on how these mechanisms develop and contribute to reading acquisition. Moreover, future work should clarify the role of attentional and reward-based mechanisms in AV learning, given their central role in perceptual learning^61,62^. Finally, it will be important to determine whether the learning difficulties observed in children with dyslexia are restricted to audiovisual processing or whether they may extend across other sensory modalities.

Taken together, these results suggest that the mechanisms supporting AV associative learning develop alongside reading expertise. As the primary constraints on reading shift from phonology-dependent processing to a more domain-general reliance on working memory mechanisms with increasing proficiency, similar patterns of mechanistic constraints may also apply to AV learning. Additionally, while we have demonstrated that a domain-general and early-developing ability to form AV associations likely makes a unique contribution to DD, we also propose that learning to read may, in turn, refine a child’s ability to acquire AV associations. However, further research with larger sample sizes is needed to validate these patterns, and in particular, to clarify the precise role of cognitive abilities in AV learning. In sum, our findings shed light on the developmental trajectory of AV associative learning, its cognitive underpinnings, and its potential role as a distinct contributor to dyslexia.

## Methods

### Study design and procedures

We recruited Italian-speaking children in two separate periods (Wave 1: 2017; Wave 2: 2024) and from two geographical regions (Wave 1: Trentino, IT; Wave 2: Ticino, CH). Of note, the data collection and analysis plan for Wave 2 was preregistered as a replication of Wave 1^48^. In addition to children with DD, both cohorts included two control groups: (i) age-matched, typically-developing children (ages 10–12) and (ii) younger, reading-matched, typically-developing children (ages 8–10). Comparisons were made based on chronological age and performance in the text reading task, respectively. This approach was crucial for avoiding the attribution of differences solely to variations in reading proficiency.

Inclusion criteria for the typically-developing groups were as follows: (i) no diagnosis of psychological/neurological disorders; (ii) no reading delay in word, nonword, and text reading tasks (not falling below −1SD from published norms); (iii) normal or corrected-to-normal vision and hearing; (iv) intelligence within the normal range (cut-off score ≥7) as measured by the Matrix reasoning subtest of WISC-IV^63,64^.

Children with DD had previously received a diagnosis of DD that followed the Italian national guidelines and was based on the International Classification of Diseases (ICD-10^65^). In addition, reading performance was required to be below 1.5 SDs (for speed) from the age-adjusted mean of the general population and/or below the fifth percentile (for accuracy) in standardized reading tests, with adequate levels of education and general intellectual abilities. No comorbid developmental disorders (e.g., ADHD, dyscalculia) were reported in this group. Children diagnosed with dyslexia were recruited from clinical centers in Trentino and Ticino, while typically-developing children were recruited from schools in the same areas.

Written informed consent was obtained from all parents after a description of the research study, in accordance with the principles of the Declaration of Helsinki. The study was approved by the research ethics committee of the University of Trento (Protocol_2017-030).

Below, we outline the differences between the two study cohorts.

Sample size was determined by feasibility constraints linked to the availability of the clinical population. As specified in the pre-registration for Wave 2, our goal was to recruit as many children with DD as possible, and to approximately match their numbers in the two control groups^48^.

Wave 1: eighty-two native Italian-speaking children participated in this behavioral study. Six children were excluded due to low performance in IQ tests (*N* = 2), word and nonword reading tests (*N* = 3), and text reading tests (*N* = 1). Additionally, one participant was excluded due to missing data in the AV task. The final sample comprised 70 children: 23 DD (mean_age_ = 11.97, SD_age_ = 0.81, *n*_female_ = 10), 22 Reading-matched (mean_age_ = 9.11, SD_age_ = 0.59, *n*_female_ = 10), and 25 Age-matched (mean_age_ = 11.79, SD_age_ = 0.36, *n*_female_ = 11). Participants were tested individually in one single session lasting 1.5 h. The AV task was administered after a short break (3–5 min) in the middle of the assessment session.

Wave 2: 87 native Italian-speaking children were recruited, and none were excluded from the study. The final sample included 29 DD (mean_age_ = 11.87, SD_age_ = 0.81, *n*_female_ = 13), 29 Reading-matched (mean_age_ = 8.98, SD_age_ = 0.58, *n*_female_ = 14), and 29 Age-matched (mean_age_ = 12.00, SD_age_ = 0.70, *n*_female_ = 17). Participants were tested individually across two sessions, each lasting 45 min, conducted on two separate days within the same week. The AV task was administered at the beginning of the second session.

### Measures

In addition to the AV associative learning task, our comprehensive battery of tasks encompassed assessments of reading skills, reading-related skills such as phonological awareness and RAN, as well as cognitive skills, including auditory and visual attention, and short-term and working memory skills. The AV task was administered on a 13″ screen, and headphones were used. The specific tasks administered to the participants are detailed below.

A gamified non-linguistic AV associative learning task was developed specifically for this study, adapted from an adult version of the same task^43^. The primary goal was for children to learn associations between pairs of unfamiliar auditory stimuli (eight environmental sounds) and novel visual symbols (three in total), as depicted in Fig. 1A (full set available in the OSF repository). Since participants had no prior knowledge of these arbitrary associations, they all started at chance level, ensuring a uniform starting point for all subjects in our modeling. The task was presented as a game to engage children, with a storyline involving three bears building a balloon for their journey around the world. Children had to learn each bear’s name by associating sequences of two auditory stimuli with the single bears. A similar, simplified version for preschool children is described in ref. ^60^.

The task administered here consisted of three main parts: two familiarization phases and the main AV learning task, which together lasted approximately 35 min. First, during auditory familiarization, the child passively listened to our unknown, meaningless auditory stimuli presented twice. Each sequence consisted of two environmental sounds (total duration: 3850 ms) with a 5-s inter-stimulus interval. These sounds were deliberately selected to be non-linguistic, difficult to label verbally, and unlike everyday environmental noises (e.g., not typical household or nature sounds), to minimize prior familiarity. As shown in previous studies using similar stimuli^66^, they were easily distinguishable despite their novelty. In line with the storyline, the child was instructed to listen to the bears’ “strange names.” The auditory familiarization was kept passive to reduce task demands at the start of the session and to avoid introducing task-specific strategies for the subsequent associative learning phase.

Second, during visual familiarization, the child familiarized with unknown visual symbols through a 1-back task, pressing a button whenever a bear with the same symbol appeared twice in a row. The symbols were adapted from the Bamum alphabet, chosen specifically to ensure they were unknown to participants and to minimize overlap with familiar letters from the Latin alphabet. The bears differed only by the symbols they displayed (see Fig. 1A). Each image was shown for 2.5 s, with a 1-s inter-stimulus interval. In total, 33 pictures were displayed, with six 1-back repetitions (two for each symbol). The sequence of pictures was identical for all participants.

After the two familiarization phases, children proceeded to the AV association task (see Fig. 1B for the trial structure). This task was divided into 6 blocks, each consisting of 12 trials, making a total of 72 trials. Visual feedback, in the form of coloring parts of a hot air balloon, was provided at the end of each block, allowing for optional breaks.

During each trial, a sequence of two auditory stimuli (selected from a set of four environmental sounds) was played without an inter-stimulus interval, while a small musical note was displayed on the screen. Children learned to associate four sound combinations (e.g., sounds 1 + 2, 3 + 2, 1 + 4, 3 + 4) with three visual symbols (bears). Two combinations (1 + 2 and 3 + 2) required attending to both sounds in the sequence, whereas the other two (1 + 4 and 3 + 4) could be solved by focusing only on the last sound (see Fig. 1A).

Following the auditory stimuli, there was a 1-s blank screen. Then, three response options (shown in Fig. 1B) were displayed until the child responded or for a maximum of 6 s. The locations of the bears changed from one trial to the next to prevent spatial learning, and children were informed of this variability during task instructions. Feedback was provided after each response, with the correct bear displaying either joy or sadness depending on whether it had been chosen, and the corresponding sounds were played again (duration: 4 s).

The AV pairings were fixed across all participants, ensuring everyone learned the same associations. However, for Wave 1, the order of presentation was randomized, differing between children. In contrast, Wave 2 had two specific orders of presentation (A and B), with half of the participants following each order.

Moreover, the task presented a scaffolding structure to introduce stimuli incrementally over the course of successive blocks, ensuring that participants could build a robust foundation before encountering the full set of stimuli. In Block 1, participants were introduced to two pairs of auditory stimuli: sound 1 + 2 and sound 3 + 4, with each pair presented six times. In Block 2, a third pair of auditory stimuli (sound 3 + 2) was introduced, bringing the total to three pairs, and enforcing attention to the sound sequence. Each pair in Block 2 was presented four times, allowing participants to start integrating the new stimuli. Starting from Block 3, the fourth pair of auditory stimuli was introduced, which mapped to an already associated visual response. Each pair was presented three times per block. Blocks 4, 5, and 6 continued with the presentation of all four AV pairs.

Standardized cognitive and linguistic tasks assessing attentional control, working memory, reading, and reading-related skills were used. The raw data were converted to *z*-scores according to published and standardized age and/or schooling-related norms (with the exception of IQ score, which was expressed as a standard score with a mean of 10 and standard deviation of 3, and the Test for Reception of Grammar^67^, which was expressed as a standard score with a mean of 100 and standard deviation of 15).

To assess children’s nonverbal reasoning abilities, the Matrices Reasoning test from the Wechsler Intelligence Scale for Children-Fourth Edition was administered^63^. In this task, the child is presented with an incomplete matrix composed of pictures or abstract drawings. The child must select the missing element from four or five response options. This task is designed to measure fluid intelligence, specifically evaluating the child’s ability to identify patterns and logical relationships among the matrix elements.

Attentional control in the visuospatial modality was evaluated using two barrage tests: the Italian version of the Bells Test^68^ and another timed cancellation task from the “Measures for Executive Attention” battery (MEA; Benso et al.^69^). In the first barrage task, the Modified Bells Test^70^, the child is presented with four sheets of paper, each containing numerous black silhouettes of familiar figures such as houses and horses. In each sheet, the child’s task is to find and cross out as many target images (35 bells) as possible within 120 s. The number of targets identified at both 30-s and 120-s intervals was recorded and used for further analysis. In the MEA cancellation task, children locate and mark target stimuli (vertical or diagonal bars) within a 45-s time limit across 10 sheets, including 6 subtests, 2 practice tasks, and 2 distractor tasks. This task provides accuracy scores for both simple and complex cancellations, with the latter involving more crowded sheets. For visual attention, a composite score was calculated from four scores: two from the Bells Test (targets at 30 and 120 s) and two from the MEA cancellation task (simple and complex cancellations).

Moreover, the Auditory Attention and Response Set subtests from the NEPSY-II^71^ (Italian version^72^) were administered. Specifically, the Auditory Attention subtest primarily measures sustained attention, that is, a child’s ability to maintain focus on auditory stimuli over an extended period. In this task, the child listens to a prerecorded list of words and must touch the appropriate circle in the stimulus book when a target word is heard. The Response Set subtest evaluates cognitive flexibility and inhibitory control. In this task, the child listens to a series of spoken instructions that require them to switch between different response sets. This necessitates inhibiting automatic responses and adapting to new instructions, thus evaluating the child’s ability to shift attention and control impulses. In both subtests, points are awarded only if the child responds correctly within 2 s of hearing the target word. Performance is evaluated through separate scores for commission errors (incorrect responses), omission errors (missed target words), and inhibitory errors (inappropriate responses). Two separate composite measures were calculated for Auditory Attention and Cognitive Flexibility by using the three relevant scores (commission errors, omission errors, and inhibitory errors), respectively.

The Digit Span subtest from the “BVS-CORSI” (Battery for the Assessment of Visual and Spatial Memory) evaluates verbal short-term memory and working memory^73^. Participants hear a sequence of digits and must recall them in the same order (Digit Forward) and in reverse order (Digit Backward). The sequences increase in length with each trial. Each correct sequence earns one point. Testing starts with Digit Forward; as soon as both trials of a sequence are incorrect, the examiner moves to Digit Backward. Digit Forward and Digit Backward were combined to calculate the Verbal Working Memory composite score.

The Corsi Block-Tapping Test evaluates visual working memory (BVS-CORSI^73^). It involves a board with nine irregularly arranged cubes, numbered on the side facing the examiner. This task involves reproducing sequences of tapped cubes in both forward and backward orders (Corsi Forward and Corsi Backward). Analogous to Digit Span, a composite score for Visual Working Memory was calculated by combining Corsi Forward and Corsi Backward scores.

The Test for Reception of Grammar-Version 2 (TROG-2^67^) is a standardized assessment designed to evaluate a child’s understanding of grammatical contrasts and thus syntactic comprehension. The test consists of 80 items, each presented as a sentence read aloud by the examiner. For each sentence, the child is shown four images and must select the one that best represents the sentence. The test covers a wide range of grammatical structures, assessing the child’s ability to comprehend various aspects of syntax and morphology.

The Phonological Processing subtest from the NEPSY-II^71^ (Italian version^72^) assesses phonemic awareness in children through two tasks: Word Segment Recognition and Phonological Segmentation. In Word Segment Recognition, the child is asked to identify a picture corresponding to a spoken word. For example, the child may be asked to identify the word “rat” by listening to the word segments “r-at” and matching it with the correct picture. Phonological Segmentation, on the other hand, requires the child to manipulate sounds in words. The child creates a new word by omitting a syllable or phoneme, or by substituting one phoneme with another. For instance, the child might be asked to say “sound,” then say the same word but replace the /s/ sound with /hə/, forming the word “hound.” Administration adjustments include changes to starting points, reverse rules, and an increased discontinue rule to six consecutive scores of 0. These tasks collectively contribute to a single standardized score representing overall phonological processing ability.

Regarding RAN, the Color Naming task (MEA^69^) was used, which assesses lexical access ability and the efficiency of attentional-executive systems. In this task, the child is presented with a sheet displaying 35 colored circles arranged in five rows. The circles are colored white, yellow, red, green, and blue. The child’s task is to name the colors of the circles as quickly and accurately as possible.

Reading skills were evaluated using two distinct tests, one assessing words and nonwords reading and the other evaluating text reading. The first task is part of the “Batteria per la valutazione della dislessia e disortografia evolutiva” (Battery for the Assessment of Developmental Reading and Spelling Disorders-2^74^). Children were asked to read aloud four lists of 28 words and three lists of 16 nonwords. Nonwords are defined as sequences of up to four syllables that do not correspond to actual words in Italian but follow legal phonetic patterns. Speed (in seconds) and errors (each incorrect word or nonword counted as one error) were measured, and *z*-scores were computed separately for word and nonword reading. These were then combined to create distinct composite scores for word reading and pseudo-word reading, respectively.

The text reading task is part of the “Batteria per la Valutazione Neuropsicologica per l’età evolutiva – BVN” (Battery for Neuropsychological Assessment^75^). The child is asked to read aloud a short text passage as quickly and accurately as possible. The final score, transformed into a *z*-score, corresponds to the number of correct syllables read within 1 min.

### Data analyses

Statistical analyses were performed in R (R Core Team^76^). Participants were excluded if, by the last 2 blocks of the learning task, their performance was not significantly above the guessing rate of 0.333 (tested using a one-tailed binomial test; for pre-registration see ref. ^48^). This procedure led to 5 excluded participants in Wave 1 (2 Dyslexic, 3 Reading-Matched, and 0 Age-Matched). No participants were excluded from Wave 2.

We fit models of trial-to-trial learning, in the form of accuracy being an exponential function of time on task^45,77,78^. This characterization of incremental learning accumulating over trials and saturating with time allows for a theoretically-guided approach to using all data simultaneously in inferring asymptotic performance and learning rate, while also controlling for other factors such as specific stimuli’s difficulty. We do note that a choice to examine group differences in accuracy on only the last three blocks, an ad hoc division of the data, would provide similar inferences (i.e., a generalized linear mixed-effects model would show that both the Reading-Matched and the Age-Matched groups had higher accuracy than the DD group).

Previous work using a similar behavioral task was well-fit using theoretically-motivated continuous-time methods^43^. We leveraged the a priori guessing rate of 1/3 to establish that all participants’ accuracies began at that value, and then estimated increases in accuracy with subsequent trials. In brief, these learning trajectories included fixed-effects estimates of asymptotic accuracy as well as the time taken to learn (“rate”). Asymptotic accuracy is reported on a logit scale, while time taken to learn is reported on a base-2 log scale.

Unlike in previous work^43^, however, the current task included an incremental introduction of stimuli. Due to the fact that novel stimuli were introduced at the beginning of the second and the third blocks of the task, in our estimation of learning trajectories we included a linearly-decaying offset from the exponential learning trajectory; this offset began at the first trial of each of these blocks and decayed to 0 by the end of that block (see Figs. S1 and S2 for characteristic shapes of these learning trajectories). As such, the coefficient estimated for this offset indicates the change (e.g., decrease in performance) associated with the introduction of a novel stimulus in that block.

Due to the limited amount of data, we included only a single parameter of between-participant differences in learning, that is, the rate of learning. We also fit a separate analogous model with asymptotic accuracy being the sole between-participant parameter, but this alternative asymptote-varying model showed poorer goodness-of-fit than our primary rate-varying model. As such, we continued by interpreting the results only of the model allowing for between-participant differences in the time taken to learn.

Due to the possibility of our samples being systematically different than published norms (e.g., our typically-developing children not having a mean matching the mean of published norms), we additionally chose to normalize performance within our samples in order to easily identify whether our DD samples showed systematic differences from our typically-developing samples. Each task outcome variable was first Yeo-Johnson transformed to minimize univariate skew across all groups and waves. Next, the results were combined to produce the following indices of performance: nonverbal reasoning, auditory attention (subscores of omission; commission; inhibition), visual attention (first task: total number of targets identified in the 30-s interval and total number of targets identified in the 120-s interval; second task: accuracy in simple cancellations and accuracy in complex cancellations), cognitive flexibility (subscores of omission; commission; inhibition), verbal working memory (forward span; backward digit span), visual working memory (corsi forward; corsi backward), syntactic comprehension, phonological awareness, RAN, word reading (word reading speed; word reading accuracy), nonword reading (nonword reading speed; nonword reading accuracy), and text reading. Where an index had only one measure, that measure was scaled such that the typically-developing children across both age-matched and reading-matched groups would have a mean of zero and standard deviation of one. Where the index had more than one measure, we used principal components analysis (PCA) on the typically-developing children’s data to determine the latent dimension explaining the most variance in the measures (i.e., the first component). We then used the component loadings for each measure, along with each participant’s score on each measure, to determine each participant’s value for the index in question. Composite scores for each of the following cognitive skills—Auditory Attention, Visual Attention, Cognitive Flexibility, Visual Working Memory, and Verbal Working Memory—were calculated by extracting the dominant first component from a PCA based on the two or three relevant scores for each skill. The composite reflected the primary dimension that explained the largest proportion of variance across the measures assessed within each respective domain. See Fig. 4 for the resulting distribution of indices; note that the DD group has reliably lower-than-typical values on the expected measures. Our typically-developing samples also indicate some divergence from standardized norms, as evidenced by the two groups appearing to perform better or worse than one another within this normalized space; these differences may be due to various unknown factors, such as sampling noise or differences in the quality of the educational system at different ages, and we do not attempt to interpret the pattern of differences.

## Supplementary information

Pasqualotto_AVL_SI_rev_final.

## Data Availability

The data are publicly available on OSF: DOI 10.17605/OSF.IO/7E2MW.

## Code availability

The code supporting the findings of this study, and necessary to replicate analyses, is included within the file entitled “Learning model” on OSF: DOI 10.17605/OSF.IO/7E2MW.
