# Supplementary material for: Impaired audio-visual associations in dyslexia: evidence beyond linguistic processing
Source: NPJ Sci Learn. 2025 Dec 17;11:1. doi: 10.1038/s41539-025-00382-7 (PMC12764848; doi:10.1038/s41539-025-00382-7)

**Impaired Audio-Visual Associations in Dyslexia: Evidence Beyond Linguistic Processing**

**SUPPLEMENTARY INFORMATION**

**1. Model specifications**

Model specifications are written in R code “Wilkensen” format, as would be used in nonlinear models fit using the package **brms**.

### **Single-cohort model**

resp_corr_b ~ groupOffsetLogit + pAsymIntercept + (0.3333333 - (pAsymIntercept)) * 2^((1 - totalTrialNum)/(pRateIntercept))

pRateIntercept ~ 2^(2 + pRate)

pRate ~ group_label + (1 | participant)

pAsymIntercept ~ inv_logit(pAsym)

pAsym ~ group_label

groupOffsetLogit ~ 2 * inv_logit(groupOffset) - 1

groupOffset ~ 0 + begin_bl2:group_label + begin_bl3:group_label + (1 | sounds)

### **Combined-cohorts model**

resp_corr_b ~ groupOffsetLogit + pAsymIntercept + (0.3333333 - (pAsymIntercept)) * 2^((1 - totalTrialNum)/(pRateIntercept))

pRateIntercept ~ 2^(2 + pRate)

pRate ~ group_label * cohort_c + (1 | participant)

pAsymIntercept ~ inv_logit(pAsym)

pAsym ~ group_label * cohort_c

groupOffsetLogit ~ 2 * inv_logit(groupOffset) - 1

groupOffset ~ 0 + begin_bl2:group_label + begin_bl2:group_label:cohort_c + begin_bl3:group_label + begin_bl3:group_label:cohort_c + (cohort_c || sounds)

*Note: this model includes main effects and interactions of a binary “cohort_c” variable which coded the first cohort as -.5 and the second as +.5, thereby leading the effects of interest (e.g., comparisons between DD and matched groups) to be at an intermediate level. Several different ways of specifying these main effects and interactions were tested to ensure robustness, and all fit models resulted in the same effects of interest being reliable.*

**2. Results**

**Wave 1 model results**

*Figure S1. Group-level results of learning model fit to data from Wave 1. Both learning (nonlinear trend) and between-block offsets (linear trend beginning during blocks 2 and 3) are shown. Error bands are not shown in order to avoid obscuring the overall trends; for indications of reliability of effects see Table S1.*


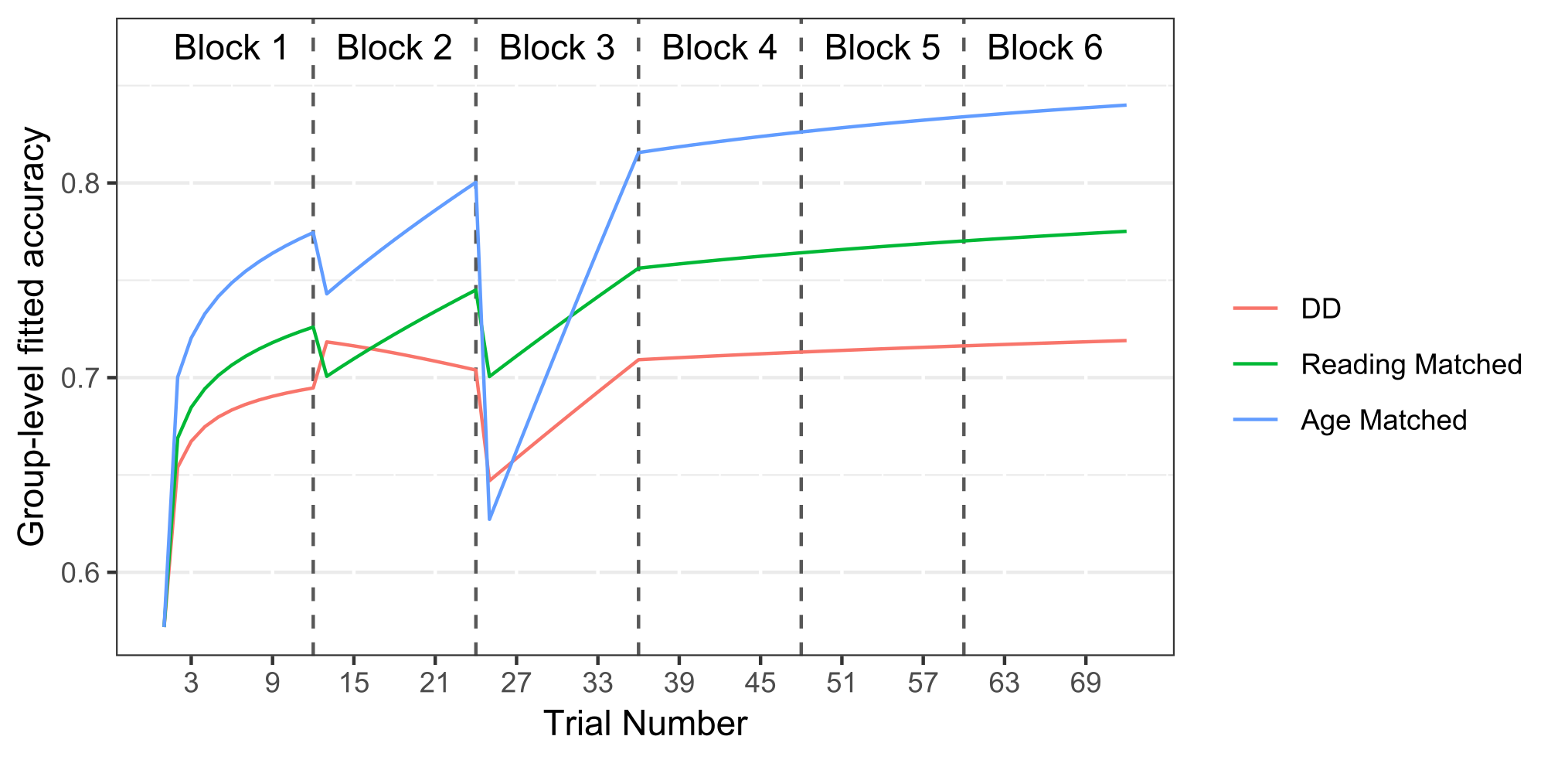


*Table S1. Fixed-effect coefficients of model fit to Wave 1. Note that the coefficients related to the nonlinear learning trajectories, Time-to-learn and Asymptote, have the DD group as a reference group; in contrast, the Block 2 offset and Block 3 offset coefficients are each in contrast to zero rather than contrasted to a reference group.*

|  | Estimate | l-95% CI | u-95% CI | reliable |
| --- | --- | --- | --- | --- |
| Time-to-learn (DD) | 2.647 | 0.397 | 5.055 | N/A |
| Time-to-learn (Age Matched – DD) | -1.662 | -4.441 | 1.074 |  |
| Time-to-learn (Reading Matched – DD) | -0.268 | -3.265 | 2.625 |  |
| Asymptote (DD) | 0.396 | 0.230 | 0.578 | N/A |
| Asymptote (Age Matched – DD) | 0.363 | 0.171 | 0.546 | * |
| Asymptote (Reading Matched – DD) | 0.254 | 0.058 | 0.437 | * |
| DD: Block 2 offset | 0.049 | -0.146 | 0.230 |  |
| Age Matched: Block 2 offset | -0.072 | -0.229 | 0.051 |  |
| Reading Matched: Block 2 offset | -0.057 | -0.248 | 0.097 |  |
| DD: Block 3 offset | -0.120 | -0.297 | 0.061 |  |
| Age Matched: Block 3 offset | -0.359 | -0.537 | -0.184 | * |
| Reading Matched: Block 3 offset | -0.095 | -0.294 | 0.069 |  |

**Wave 2 model results**

*Figure S2. Group-level results of learning model fit to data from Wave 2. Both learning (nonlinear trend) and between-block offsets (linear trend beginning during blocks 2 and 3) are shown. Error bands are not shown in order to avoid obscuring the overall trends; for indications of reliability of effects see Table S2.*


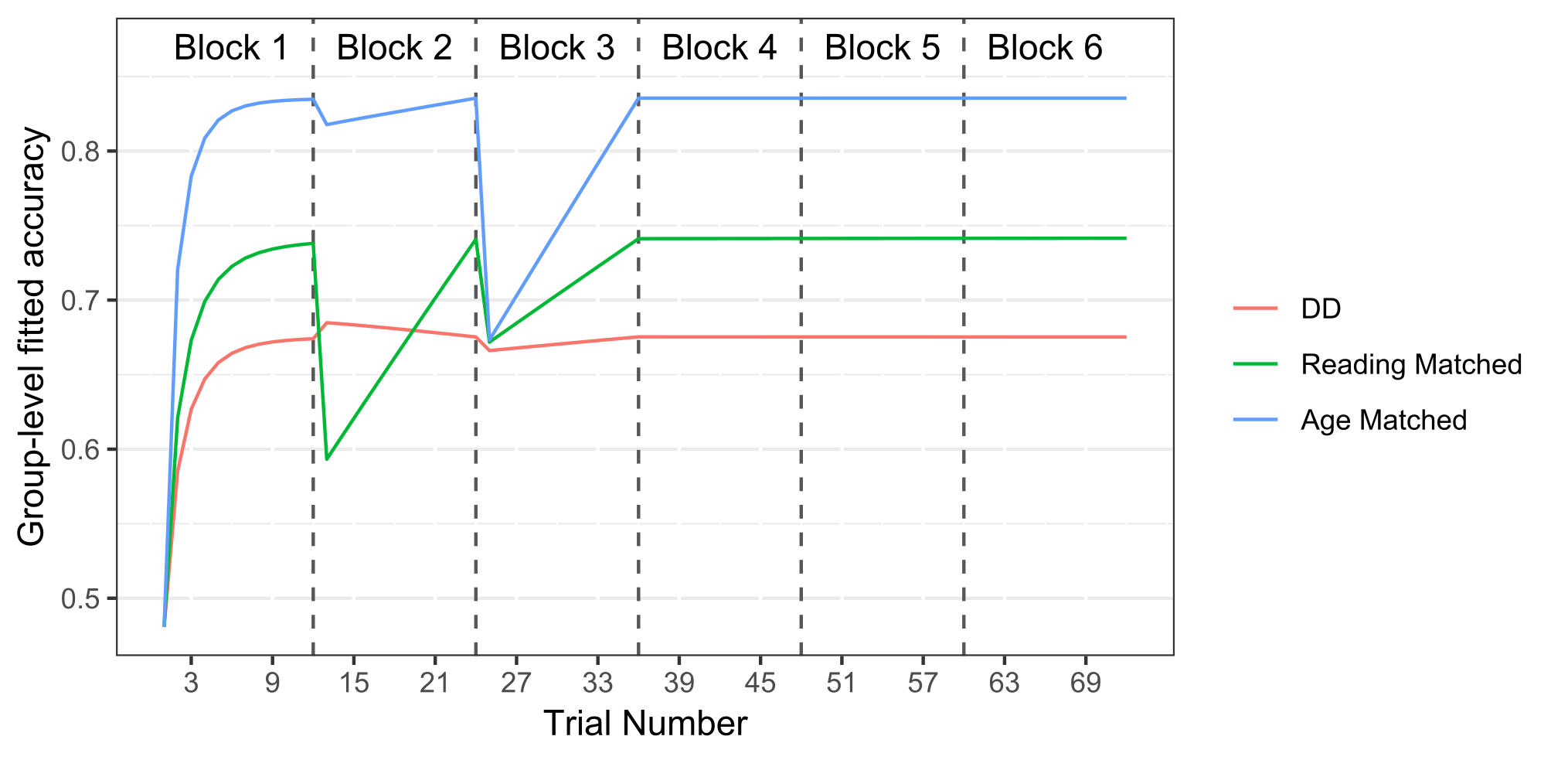


*Table S2. Fixed-effect coefficients of model fit to Wave 2. Note that the coefficients related to the nonlinear learning trajectories, Time-to-learn and Asymptote, have the DD group as a reference group; in contrast, the Block 2 offset and Block 3 offset coefficients are each in contrast to zero rather than contrasted to a reference group.*

|  | Estimate | l-95% CI | u-95% CI | reliable |
| --- | --- | --- | --- | --- |
| Time-to-learn (DD) | -1.917 | -3.476 | 0.105 | N/A |
| Time-to-learn (Age Matched – DD) | -0.935 | -3.925 | 0.965 |  |
| Time-to-learn (Reading Matched – DD) | -0.145 | -2.747 | 1.772 |  |
| Asymptote (DD) | 0.114 | -0.590 | 0.743 | N/A |
| Asymptote (Age Matched – DD) | 0.706 | 0.541 | 0.932 | * |
| Asymptote (Reading Matched – DD) | 0.280 | 0.136 | 0.436 | * |
| DD: Block 2 offset | 0.022 | -0.161 | 0.202 |  |
| Age Matched: Block 2 offset | -0.035 | -0.182 | 0.111 |  |
| Reading Matched: Block 2 offset | -0.295 | -0.482 | -0.113 | * |
| DD: Block 3 offset | -0.018 | -0.210 | 0.164 |  |
| Age Matched: Block 3 offset | -0.329 | -0.499 | -0.160 | * |
| Reading Matched: Block 3 offset | -0.141 | -0.329 | 0.038 |  |

**Performance in cognitive, reading and reading-related tasks as well as their associations with AV learning – Separated by wave**

*Figure S3. Spearman correlations with non-learning measures, separated by wave (horizontal panels).*


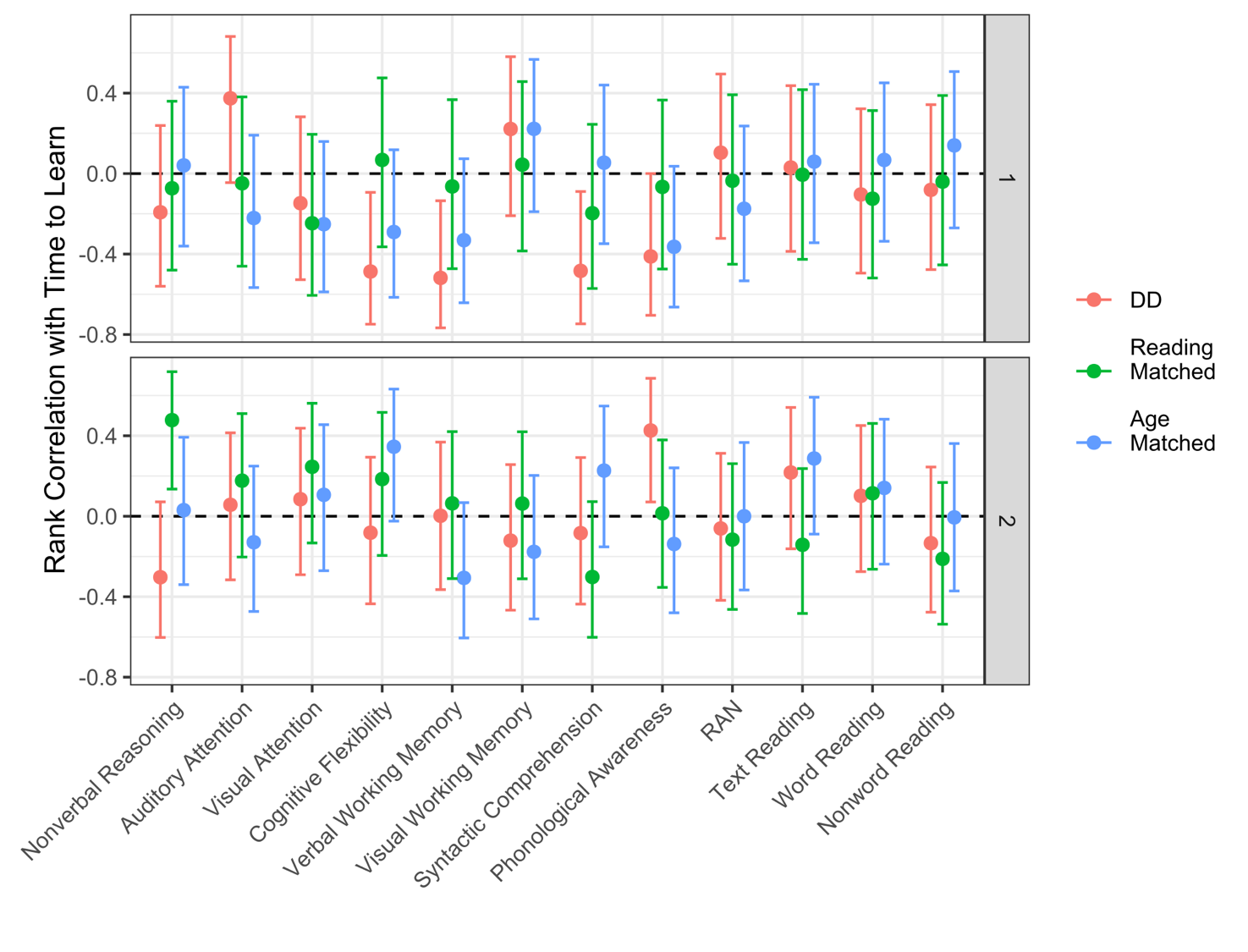


*Figure S4. Composite scores per group. Horizontal panels indicate wave number.*


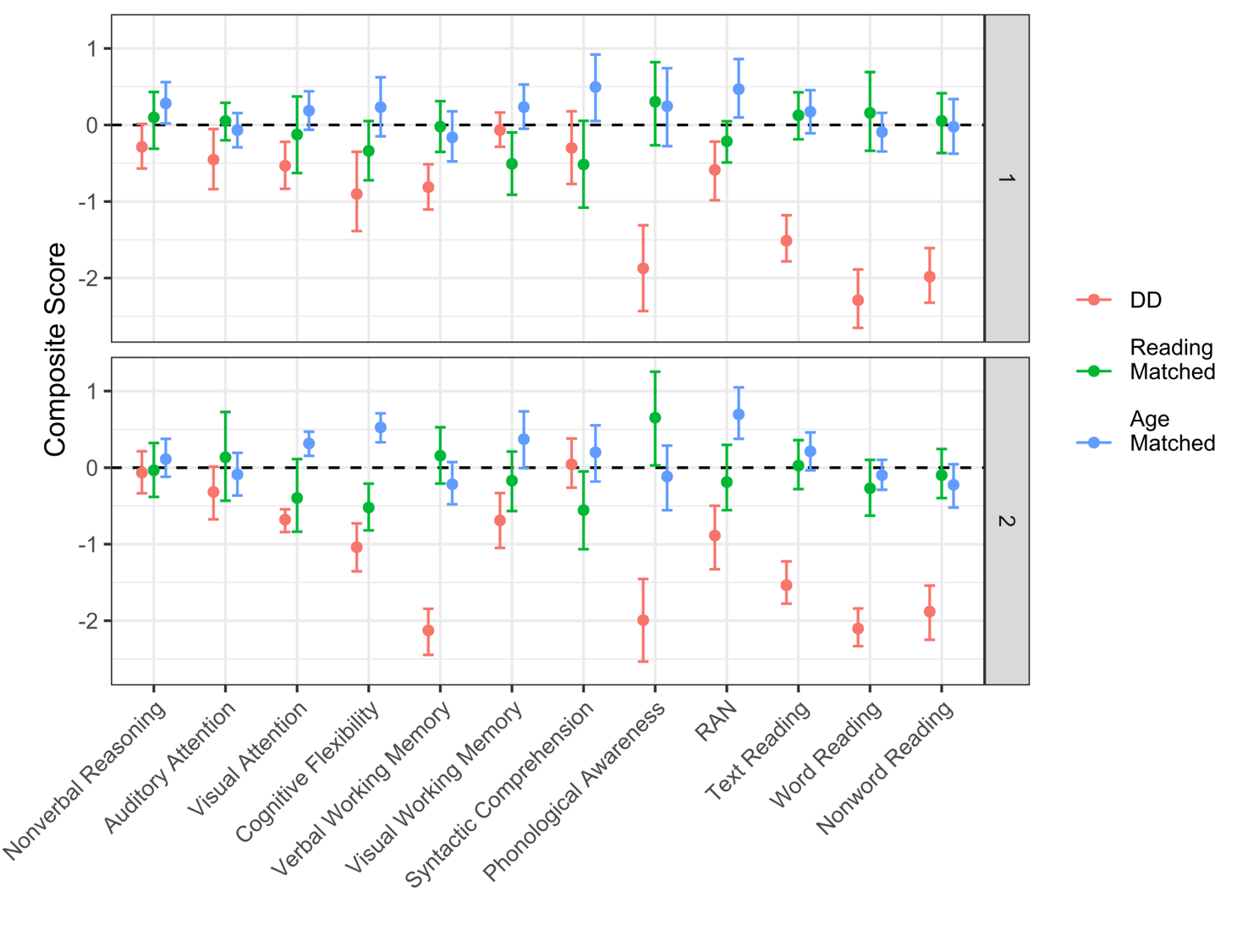

Supplement: Supplementary file 1 — Pasqualotto_AVL_SI_rev_final. [file 41539_2025_382_MOESM1_ESM.docx]
